# Impaired kidney function at ED admission: a comparison of bleeding complications of patients with different oral anticoagulants

**DOI:** 10.1186/s12873-021-00497-1

**Published:** 2021-09-18

**Authors:** Martin Müller, Michaela Traschitzger, Michael Nagler, Spyridon Arampatzis, Aristomenis K. Exadaktylos, Thomas C. Sauter

**Affiliations:** 1 Department of Emergency Medicine, Inselspital, Bern University Hospital, Bern University, Bern, Switzerland; 2 University Institute of Clinical Chemistry, Inselspital Bern University Hospital, and University of Bern, Bern University, Bern, Switzerland; 3 Department of Nephrology and Hypertension, Inselspital, Bern University Hospital, University of Bern, Bern, Switzerland

**Keywords:** Anticoagulants, Bleeding, Direct oral anticoagulants, Kidney function, Vitamin-K antagonist

## Abstract

**Background:**

Up to a fourth of patients at emergency department (ED) presentation suffer from acute deterioration of renal function, which is an important risk factor for bleeding events in patients on oral anticoagulation therapy. We hypothesized that outcomes of patients, bleeding characteristics, therapy, and outcome differ between direct oral anticoagulants (DOACs) and vitamin-K antagonists (VKAs).

**Methods:**

All anticoagulated patients older than 17 years with an impaired kidney function treated for an acute haemorrhage in a large Swiss university ED from 01.06.2012 to 01.07.2017 were included in this retrospective cohort study. Patient, treatment, and bleeding characteristics as well as outcomes (length of stay ED, intensive care unit and in-hospital admission, ED resource consumption, in-hospital mortality) were compared between patients on DOAC or VKA anticoagulant.

**Results:**

In total, 158 patients on DOAC and 419 patients on VKA with acute bleeding and impaired renal function were included. The renal function in patients on VKA was significantly worse compared to patients on DOAC (VKA: median 141 μmol/L vs. DOAC 132 μmol/L, *p* = 0.002). Patients on DOAC presented with a smaller number of intracranial bleeding compared to VKA (14.6% DOAC vs. 22.4% VKA, *p* = 0.036). DOAC patients needed more emergency endoscopies (15.8% DOAC vs, 9.1% VKA, *p* = 0.020) but less interventional emergency therapies to stop the bleeding (13.9% DOAC vs. 22.2% VKA, *p* = 0.027). Investigated outcomes did not differ significantly between the two groups.

**Conclusions:**

DOAC patients were found to have a smaller proportional incidence of intracranial bleedings, needed more emergency endoscopies but less often interventional therapy compared to patients on VKA. Adapted treatment algorithms are a potential target to improve care in patients with DOAC.

## Background

Any type of anticoagulation is associated with the risk of bleeding complications [1]. Frequency of bleeding and bleeding locations, however, may differ among different classes of anticoagulants. Intracranial haemorrhage and gastrointestinal bleedings (GIB) are the most common bleeding complications leading to major haemorrhage in patients on direct oral anticoagulants (DOAC) therapy [2]. Compared to classic vitamin K antagonist therapy (VKA), DOAC were found to be associated with a 50% reduction of intracranial haemorrhage [3, 4]. In contrast to intracranial haemorrhage, the incidence of GIB in DOAC patients is at least comparable to VKA or may be even higher for dabigatran and rivaroxaban [5].

Treatment of special patient groups with the need of anticoagulation e.g. patients with chronic kidney diseases (CKD) or at risk for CKD [2, 6] may be more demanding. Patients with atrial fibrillation and CKD are a special treatment challenge, because they combine an increasing risk of both, bleeding in general and thrombotic complications [7, 8]. A previous study found major bleeding events in elderly patients on DOAC therapy to be associated with a decline of renal function [9]. All registered DOAC are eliminated to a certain extent through the kidneys and thus all have limitations in patients with CKD [7]. However, recent clinical trials suggest that DOAC may be also beneficial in comparison to VKA in CKD patients [4, 10].

Current clinical guidelines recommend careful considerations about the risk and benefit in patients with impaired kidney function and need for anticoagulation [11] and specifically emphasis on the need of a stable situation to assess the kidney function and warn not to confuse acute renal impairment with CKD [7]. In contrast to this premise of a “stable situation”, up to a fourth of patients with whom an emergency physician is confronted at emergency department (ED) admission with an acute medical problem suffer from acute deterioration of renal function with unclear dimensions and dynamics [12]. This acute influence of situational environmental factors is difficult to represent in classical RCTs, which is why real-world data is needed for this purpose [13, 14].

Therefore, our primary study aim is to analyse bleeding and patient characteristics in patients with impaired kidney function at ED admission due to acute haemorrhage on DOAC therapy and compare them to patients on VKA. Furthermore, we investigate clinical outcomes (in-hospital mortality) and procedural outcomes, i.e. length of stay in the ED and in hospital, intensive care unit (ICU) transmission and hospitalisation rate as well as ED resource consumption, all compared to patients on VKA.

## Methods

### Study design & setting

This is a retrospective cohort study of the adult ED of the Bern University Hospital, Inselspital, Switzerland. Our ED is responsible for the emergency treatment of about 50,000 patients per year from a catchment area of 2 million people.

### Eligibility criteria

#### Inclusion criteria

This study included all patients treated for acute haemorrhage at our ED from 1st of June, 2012 to 30th of June, 2017 with i) VKA or DOAC therapy for full anticoagulation and ii) an impaired kidney function at admission defined as an glomerular filtration rate (GFR) ≤60 mL/min [15].

#### Exclusion criteria

The exclusion criteria were i) no creatinine testing performed at ED admission, ii) GFR > 60 mL/min at admission, iii) no oral anticoagulation medication documented in the medical report, and iv) no acute bleeding complication at ED admission.

### Primary and secondary outcomes

Primary study outcomes were bleeding and patient characteristics on DOAC therapy compared to VKA.

Secondary outcomes were procedural outcomes (length of stay in the ED and in hospital, ICU transmission and hospitalisation rate as well as ED resource consumption) and clinical outcomes, i.e. in-hospital mortality.

### Definitions

The National Kidney Foundation Kidney Disease Outcomes Quality Initiative (KDOQI) classification was used for classification of kidney impairment [15], with the severity grades according to the GFR of 45–60 mL/min, 30-44 mL/min, 15-29 mL/min, and < 15 mL/min. The GFR was calculated using the CKD-EPI equation as it was recommended by the National Kidney Foundation because of the reliability across all CKD stages [16]. Although the Cockroft-Gault equation for GFR estimation was applied in the landmark clinical trials of DOAC in atrial fibrillation, it has several limitation in its application particularly in patients that are overweight and older [7].. Since the implementation of the Cockroft-Gault equation was not feasible in our retrospective ED study as patient weight is not documented for all ED patients we performed the analysis using the CKD-EPI equation which is calculated automated in all patients. Polypharmacy was defined as the use of five or more drugs [17].

To describe comorbidities of a patient, the Charlson comorbidity index was used including parameter such as liver disease, CKD, diabetes, cerebrovascular disease, and congestive heart failure [18].

*Major bleeding* were defined for our study according to the international society of thrombosis and haemostasis (ISTH) definition as fatal bleeding, symptomatic bleeding in critical organ or transfusion of at least two erythrocytes concentrates [19]. As a documented haemoglobin drop of at least 20 g/L was not feasible to determine reliable in the ED setting, the criterion was not used.

In Switzerland, the costs incurred in an ED for each ambulatory procedure or physician/nurse work are settled in “tarmed: tarif médical” points which, is a system of procedure codes. This point system used in Switzerland corresponds to 1 Swiss franc per 1 point billed. We indicate the total ED resource consumption during treatment in “tax points” as the sum of all procedures.

### Data handling

A keyword search was performed through all medical reports between 01.06.2012 to 01.07.2017 containing all the substance classes and brand names of oral anticoagulants approved in Switzerland (substance classes: rivaroxaban, dabigatran, apixaban, and edoxaban, phenprocoumon, warfarin, and acenocoumarol), combined with the logic operator “OR”. Medical ED reports in our department including patient history, diagnosis, clinical findings, and medication administered are stored electronically in an ED patient database (E-Care, ED 2.1.3.0, Turnhout, Belgium). We excluded all search results of cases without creatinine testing at ED admission. Finally, the eligibly criteria were evaluated by manual full text analysis of each found medical ED report.

The following parameter were extracted directly from the database: age, sex, triage level (which is routinely determined by special trained nurses using the Swiss triage scale [20]), type of referral, all procedural outcomes, in-hospital mortality, as well as creatinine and INR values on admission. Using full-text analysis of the medical report, the following parameter were manually extracted: trauma, information on anticoagulation (indication for therapy, type of medication, additional antiplatelet therapy), comorbidities to calculate the Charlson comorbidity index, arterial hypertension and polypharmacy, bleeding characteristics i.e. location and extent of bleeding (major bleeding and the discharge diagnosis of a haemorrhage shock) as well as information on the therapy of the bleeding. Interventional therapies included surgery, coiling, clipping, embolization, angiography, drainage, and variceal ligation. A diagnostic endoscopy was not considered an interventional therapy.

### Group description and data analysis

The DOAC group used for analysis combined all patients on any DOAC approved in Switzerland (apixaban, dabigatran, edoxaban and rivaroxaban) on ED admission. For group comparison we formed a VKA group, including all patients under phenprocoumon and acenocumarol at ED admission. All statistical analysis for this work was performed with Stata® 13.1 (StataCorp, The College Station, Texas, USA).

For descriptive analysis the distribution of categorical variables was presented as the absolute accompanied by the relative number in each category [n (%)] and of continuous outcomes with the median accompanied by the interquartile range (IQR) as normal distribution of continuous outcomes could not be ensured.

To compare the study groups (DOAC vs. VKA) Chi square and Wilcoxon rank sum tests were used, as appropriate. The significance level was set to *p* < 0.05. As this is an exploratory data analysis, there was no adjustment for multiple testing.

### Ethical considerations

The present study is registered with the competent ethics committee of Canton Bern, Switzerland under the number 073/2015 and according to Swiss law, the need to obtain informed consent for the study was waived.

## Results

**Fig. 1:**
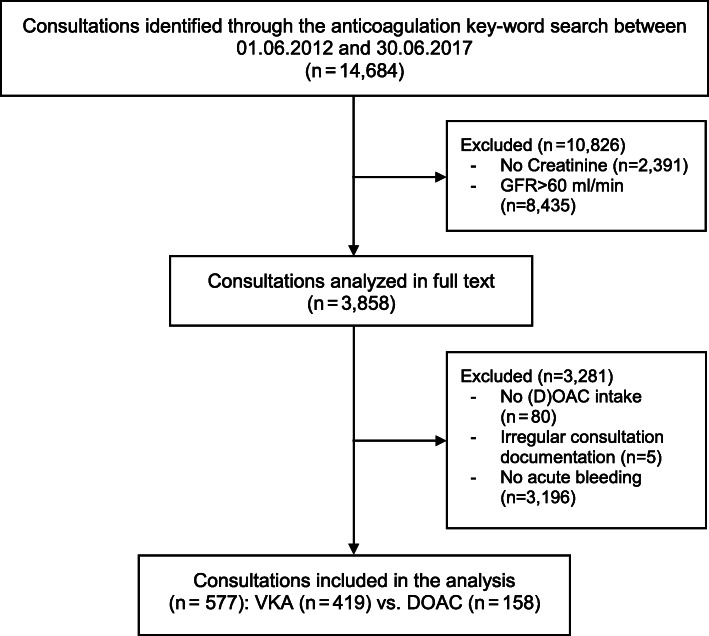
Flowchart. Abbreviations: DOAC, direct oral anticoagulant; VKA, Vitamin K antagonists

Anticoagulation key-word search between 01.06.2012 to 31.06.2017 resulted in 14,684 patients out of 199,982 consultations (Fig. 1).

In 2391 patients, no creatinine measurements were performed during ED workup and 8435 patients had a renal function above the cut-off value (GFR > 60 ml/min) and were therefore excluded.

By manual screening and exclusion of 3281 patients without an acute diagnosis of bleeding (*n* = 3196), unclear or no-anticoagulation status at ED admission (*n* = 80), and five patients with irregular consultation documentation (e.g. duplicates), we finally included 158 ED consultations on DOAC and 419 consultations on VKA. The ratio between VKA and DOAC patients decreased over the study years. While in the first year of the study (2012/2013) 91.0% of the patients were on VKA, in the last year of the study 58.2% (2016/2017) were on VKA therapy (*p* < 0.001).

### Patient characteristics

An overview of all patient characteristics is presented in Table 1.

**Table 1:** Patient characteristics

	DOAC (*n*=158)	VKA (*n*=419)	Total (*n*=577)	p
**Demographic data**				
Sex, male [n (%)]	88 (55.7)	239 (57.0)	327 (56.7)	0.771
Age, [median (IQR)]	79.0 (72-83)	79.0 (70-85)	79.0 (71-84)	0.774
**Renal function**				
Creatinine, μmol/L [median (IQR)]	132 (110-162)	141 (118-193)	138 (115-182)	0.002
GFR group, [n (%)]				
45-59 mL/min	61 (38.6)	131 (31.3)	192 (33.3)	
30-44 mL/min	65 (41.1)	147 (35.1)	212 (36.7)	
15-29 mL/min	31 (19.6)	110 (26.3)	141 (24.4)	
<15 mL/min	1 (0.6)	31 (7.4)	32 (5.5)	0.002
**Comorbidity**				
Charlson Comorbidity Index, [median (IQR)]	5 (4-7)	5 (4-7)	5 (4-7)	0.144
Diabetes, [n (%)]	45 (28.5)	123 (29.4)	168 (29.1)	0.837
Liver insufficiency, [n (%)]	9 (5.7)	9 (2.1)	18 (3.1)	0.029
Arterial hypertension, [n (%)]	109 (69.0)	306 (73.0)	415 (71.9)	0.335
Polypharmacy, [n (%)]	138 (87.3)	353 (84.2)	491 (85.1)	0.352

*Abbreviations*: *DOAC* direct oral anticoagulants, *GFR* glomerular filtration rate, *IQR* Interquartile range, *VKA* Vitamin-K antagonist

There were no significant differences regarding gender and age (*p* = 0.771 and *p* = 0.774) between both anticoagulation groups (see Table 1). The renal function in patients on VKA was significantly (*p* = 0.002) worse compared to patients on DOAC. Especially in the GFR < 15 mL/min group 7.4% patients were on VKA vs. 0.6% on DOAC. Significant more patients (*p* = 0.029) on DOAC (5.7%) had a documented liver insufficiency compared to VKA (2.1%). The Charlson comorbidity index (CCI) had a median of 5 (IQR 4–7) points in both groups (*p* = 0.143). There was no significant group difference regarding polypharmacy (*p* = 0.352).

### Consultation and anticoagulation characteristics

The type of referral or triage category was similar in both two study groups (Table 2). Trauma was found more often in VKA patients (32.9%, *n* = 138) compared to DOAC patients (24.1%, *n* = 38, *p* = 0.039). In the DOAC group, the majority of DOAC patients were on rivaroxaban (*n* = 141, 89.2%). Nine patients were on apixaban (5.7%), six on dabigatran (3.8%), and two (1.3%) on edoxaban. In the VKA group, the majority of 415 patients were on phenprocoumon (99.0%); 4 patients were on acenocumarol (1.0%). Regarding indication for anticoagulation, the most common indication for anticoagulation in all patients was atrial fibrillation (49.7%). Significant group differences were found regarding “mechanical heart valve” and “venous embolism” as indications for anticoagulation (*p* = 0.009). Additional antiplatelet therapy did not differ significantly between the two study groups (*p* = 0.346).

**Table 2:** Consultation and anticoagulation characteristics

	DOAC (*n* = 158)	VKA (*n* = 419)	Total (*n* = 577)	p
**Consultation**				
**Referral, [n (%)]**				
Ambulance	47 (29.7)	138 (32.9)	185 (32.1)	
General Practitioner	15 (9.5)	41 (9.8)	56 (9.7)	
External Hospital	32 (20.3)	75 (17.9)	107 (18.5)	
Air Rescue	0 (0.0)	8 (1.9)	8 (1.4)	
Walk-In	37 (23.4)	72 (17.2)	109 (18.9)	
Internal Referral	5 (3.2)	10 (2.4)	15 (2.6)	
No Information	22 (13.9)	75 (17.9)	97 (16.8)	0.282
**Triage, [n (%)]**				
Life-threatening	29 (18.4)	72 (17.2)	101 (17.5)	
Urgent conditions	71 (44.9)	194 (46.3)	265 (45.9)	
Semi-urgent conditions	51 (32.3)	134 (32.0)	185 (32.1)	
Non urgent conditions	3 (1.9)	8 (1.9)	11 (1.9)	
Missing	4 (2.5)	11 (2.6)	15 (2.6)	0.998
**Trauma**	38 (24.1)	138 (32.9)	176 (30.5)	0.039
**Anticoagulation**				
**Medication, [n (%)]**				
Phenprocoumon		415 (99.0)	415 (71.9)	
Acenocumarol		4 (1.0)	4 (0.7)	
Rivaroxaban	141 (89.2)		141 (24.4)	
Dabigatran	6 (3.8)		6 (1.0)	
Apixaban	9 (5.7)		9 (1.6)	
Edoxaban	2 (1.3)		2 (0.3)	< 0.001
**Reason OAK, [n (%)]**				
Mechanical valve	2 (1.3)	36 (8.6)	38 (6.6)	
Venous embolism	34 (21.5)	59 (14.1)	93 (16.1)	
Atrial fibrillation	85 (53.8)	202 (48.2)	287 (49.7)	
Combination	17 (10.8)	56 (13.4)	73 (12.7)	
Other	5 (3.2)	17 (4.1)	22 (3.8)	
Unknown	15 (9.5)	49 (11.7)	64 (11.1)	0.009
**INR**	1.3 (1.1–1.5)	2.4 (1.7–3.3)	2.0 (1.3–3.0)	< 0.001
**Antiplatelet therapy, [n (%)]**	52 (32.9)	121 (28.9)	173 (30.0)	0.346

*Abbreviations*: *DOAC* direct oral anticoagulants, *OAK* oral anticoagulation, *VKA* Vitamin-K antagonist

The median INR in patients with VKA was 2.4 (IQR: 1.7–3.3), and in patients with DOAC 1.3 (IQR 1.1.-1.5). Using the INR range 2.0–3.0 to the definition of therapeutic range, 31.3% of the VKA patients were over and 34.4% were under the therapeutic range.

### Bleeding characteristics

Apart from a higher number of intracranial bleedings including intracerebral bleedings in the VKA group (22.4% vs. 14.6% in DOAC patients, *p* = 0.036), no significant differences in the distribution of bleeding locations were found, see Table 3. The most common location was GIB in both anticoagulation groups (DOAC 34.8% vs. VKA 28.6%, *p* = 0.135). The incidence of major bleeding events did not differ significantly between the study groups (*p* = 0.784). Major bleeding was not significantly associated with the GFR in the DOAC (*p* = 0.723) respectively VKA group (*p* = 0.795).

**Table 3:** Distribution of bleeding characteristics of all included hemorrhages

	DOAC (*n* = 158)	VKA (*n* = 419)	Total (*n* = 577)	p
**Location, [n (%)]**^a^				
Epistaxis	14 (8.9)	46 (11.0)	60 (10.4)	0.457
Oral	2 (1.3)	8 (1.9)	10 (1.7)	0.597
Intracranial	23 (14.6)	94 (22.4)	117 (20.3)	0.036
Thorax	5 (3.2)	8 (1.9)	13 (2.3)	0.365
Extremity	1 (0.6)	0 (0.0)	1 (0.2)	0.103
Gastrointestinal	55 (34.8)	119 (28.4)	174 (30.2)	0.135
Intraabdominal	3 (1.9)	5 (1.2)	8 (1.4)	0.518
Retroperitoneal	1 (0.6)	10 (2.4)	11 (1.9)	0.170
Superficial	26 (16.5)	77 (18.4)	103 (17.9)	0.591
Intraocular	0 (0.0)	2 (0.5)	2 (0.3)	0.384
Intraarticular	1 (0.6)	6 (1.4)	7 (1.2)	0.434
Intraspinal	1 (0.6)	1 (0.2)	2 (0.3)	0.472
Intramuscular	4 (2.5)	25 (6.0)	29 (5.0)	0.092
Gross haematuria	18 (11.4)	34 (8.1)	52 (9.0)	0.220
Haemoptysis	3 (1.9)	7 (1.7)	10 (1.7)	0.851
Other location	22 (13.9)	41 (9.8)	63 (10.9)	0.155
**Extent of bleeding, [n (%)]**				
Haemorrhagic shock	10 (6.3)	21 (5.0)	31 (5.4)	0.531
Major bleeding	67 (42.4)	183 (43.7)	250 (43.3)	0.784

*Abbreviation*: *DOAC* direct oral anticoagulants, *VKA* Vitamin-K antagonist

^a^ the study population is by definition made of patients admitted for major hemorrhage, thus less frequent occurrence of one variable might be caused by a higher frequency of another variable and vice versa

VKA patients needed a higher number of interventions to stop the bleeding compared to DOAC (22.2% vs. 13.9%, *p* = 0.027) and a smaller number needed gastroduodenoscopies (VKA 9.1% vs. DOAC 15.8%, *p* = 0.020), see Table 4.

**Table 4:** Therapy of bleeding

	DOAC (*n* = 158)	VKA (*n* = 419)	Total (*n* = 577)	p
Therapy interventional, [n (%)]	22 (13.9)	93 (22.2)	115 (19.9)	0.027
Gastroduodenoscopy needed, [n (%)]	25 (15.8)	38 (9.1)	63 (10.9)	0.020
Colorectoscopy needed, [n (%)]	6 (3.8)	12 (2.9)	18 (3.1)	0.565
Bronchoscopy needed, [n (%)]	1 (0.6)	0 (0.0)	1 (0.2)	0.103

Abbreviations: *DOAC* direct oral anticoagulants, *VKA* Vitamin-K antagonist

### Clinical outcomes

Most patients were treated in hospital (DOAC 92.4% vs. VKA 90.2%, *p* = 0.431) and 42.0% were admitted to ICU (DOAC 38.2% vs. VKA 43.4%, *p* = 0.264) without group difference for both outcomes, see Table 5. No differences were observed regarding ED resource consumption, LOS in hospital or the ED as well as in-hospital mortality.

**Table 5:** Secondary outcomes

	DOAC (*n* = 158)	VKA (*n* = 419)	Total (*n* = 577)	p
**Procedural outcomes**				
LOS ED, hours, [median (IQR)]^a^	5.2 (3.6–7.2)	4.6 (3–6.7)	4.8 (3.1–6.8)	0.091
LOS hospital, days, [median (IQR)]	6.0 (3.1–8.9)	5.8 (2.9–10.3)	5.8 (3–9.9)	0.619
Hospitalisation, [n (%)]	146 (92.4)	378 (90.2)	524 (90.8)	0.417
ICU admission, [n (%)]	60 (38.2)	179 (43.2)	239 (41.9)	0.278
Total ED resource consumption, tax points, [median (IQR)]	1274 (708–1942)	1111 (604–1795)	1177 (632–1828)	0.091
**Clinical Outcome**				
In-hospital mortality, [n (%)]	11 (7.0)	21 (5.1)	32 (5.6)	0.370

Abbreviations: *ED* emergency department, *DOAC* direct oral anticoagulants, *IQR* interquartile range, *VKA* Vitamin-K antagonist, *LOS* length of stay. ^a^ present in 534 (92.4%) consultations

## Discussion

In this study patients with impaired kidney function and bleeding complications at ED admission, DOAC patients were found to have a lower proportional incidence of intracranial haemorrhage, needed more emergency endoscopies, but less interventional therapies to stop the bleeding compared to patients on VKA therapy.

In our study, the group of patients with VKA therapy in general had a worse renal function compared to DOAC mostly due to patients with impaired kidney function with GFR < 15 ml/min, a range with very limited experience with DOAC and contraindications for most DOAC. A relevant number of patients on DOAC therapy were found to have a renal function < 30 ml/min at admission that might potentially lead to accumulation of anticoagulant medications [21]. This finding underlines the fact that all of the DOAC have dosing regimens that need close monitoring of the renal function which have be taken into account for selection of the correct dosage.

In such patients current prescribing patterns may lead to increased thrombo-embolic risk and increased bleeding complications thus measurement of serum DOAC levels might be useful to guide acute treatment as well as further dose adjustments.

Additional platelet aggregation inhibitor therapy that could further increase the risk of bleeding and is associated with a higher and more severe bleeding risk [22, 23] was similarly distributed between the study groups.

### Bleeding location and therapy

Most of the bleeding locations occurred in a comparable frequency in both, DOAC and VKA patients with impaired renal function. GIB are a typical bleeding location for DOAC especially on rivaroxaban therapy [24]. For both anticoagulant groups, GIB were the most common bleeding leading to ED admission in our study. Intracranial bleedings were more prevalent in the VKA group. As the study population consisted of patients admitted for major hemorrhage, less frequent occurrence of one bleeding location might be caused by a higher frequency of another bleeding location and vice versa. Thus, only proportional incidences could be determined in this study.

Patients with GIB under DOAC therapy received more endoscopies overall and especially more gastroscopies compared to VKA, although VKA patients received more interventional therapies. Several reasons might explain these findings. First, physicians’ management of DOAC-associated bleedings might differ as the specific reversal agents of DOACs are expensive and may not be available in some ED. Therefore, treating physicians are more cautious as a multi-nominal survey about bleeding management demonstrated [25]. This fact is reflected by another study of our group that found an increased application of prothrombin complex concentrates for reversal of DOAC compared to VKA bleedings in clinical practice [26]. A recent meta-analysis of PCC and andexanet alfa for management of factor Xa inhibitor related bleeding, found similar rates of good hemostasis for both medications but a tendency to higher complication rates on andexanet alfa [27]. Second, several studies suggested a higher rate of GIB in DOAC patients [5, 28], thus, a wait-and-see approach might seem an inappropriate choice for the attending physician in DOAC patients with suspected GIB as the extend of the bleeding in often unclear. Third, VKA reversal is straightforward und routinely measurable compared to DOAC, thus, some interventional procedures might have been performed in VKA patients, while DOAC interventions were postponed.

### Outcomes

No significant differences were found in the investigated outcomes between the DOAC and VKA group. The hospitalisation and ICU transmission rates were high, a finding that is explained by the high number of GIB and intracranial bleedings in both groups needing close clinical monitoring or further invasive therapies.

### Limitations

Due to the limitations imposed by the size of the study and the retrospective design, there are potential confounders that were not considered in our study. Furthermore, despite the adequate documentation of complications in a tertiary hospital setting and high number of patients on both anticoagulant group, it is possible that the group sizes were still not sufficient in order to find differences in infrequent outcomes such as in-hospital mortality. Because of the monocentric design transferability to other settings is only possible to a limited extent and should be performed carefully. These limitations are not unique to our study, but rather apply to most observational pharmacoepidemiologic studies. Investigation of real-word data can be an important complement to randomized-controlled-trails, however, conclusions must be drawn with great caution due to the uncontrolled confounding factors [13, 14]. Also, we had no information on the chronicity of CKD or on the duration of the anticoagulation therapy. A single creatinine obtained during emergency admission can only provide a snapshot of the patient’s renal function. The same applies for the INR values, e.g. after reversal of VKA with PCC. Furthermore, levels of DOAC or aPTT were not measured routinely over the study period, thus the decision how to treat a patient on DOAC was often based on anamnestic data of DOAC intake. There is growing evidence, that NOAC level measurements should be standard practice, especially in at-risk populations such as patients with renal insufficiency [29].

It is well known that decisions in emergency patients have to be based on often incomplete findings and have to be made under time pressure, which does not allow the detailed collection of follow-up parameters [30–32]. In line with the local prevalence of anticoagulant medications most investigated patients were admitted on rivaroxaban [2]. Thus, separate analysis or comparison of different DOAC or stratified presentations of the outcome by GFR group was not possible due to small numbers of patients in those subgroups. With the fast adoption of DOAC including elderly patients over the study years, more DOACs were subscribed to patients with pre-existing conditions and therefore at risk for an impaired kidney function and bleedings. With our data, the exact impact on the results cannot be stated. However, in our opinion, the main findings of a lower proportional incidence of intracranial haemorrhage, more emergency endoscopies and less interventional therapies in DOAC patients remain untouched by this finding.

## Conclusion

Compared to VKA, emergency admissions due to acute haemorrhage on anticoagulation therapy with impaired renal function on presentation on DOAC therapy had a lower incidence of intracranial haemorrhage. GIB in DOAC patients needed a higher number of gastroduodenoscopies but less interventional therapies to stop the bleeding compared to VKA therapy.

Further prospective multi-centre research in the special patient population of patient with impaired renal function including DOAC level measurements is necessary. Recent developments with specific antidotes and rapidly and readily available level measurements may optimize future therapy in this special patient population.

## Data Availability

For interested researchers the analysed dataset is available from the corresponding author according to Swiss law.

## Abbreviations

CKD: chronic kidney disease
DOAC: direct oral anticoagulant
ED: emergency department
GFR: glomerular filtration rate
ICU: intensive care unit
IQR: interquartile range
LOS: length of stay
VKA: vitamin K antagonists
